# Optimal harvest-time to maximize the annual camptothecin production by *Ophiorrhiza pumila* in a plant factory with artificial light

**DOI:** 10.1007/s11418-022-01634-1

**Published:** 2022-07-05

**Authors:** Ji-Yoon Lee, Eiji Goto, Hideo Yoshida, Shoko Hikosaka

**Affiliations:** 1 Graduate School of Horticulture, Chiba University, Matsudo, 271-8510 Japan; 2 Graduate School of Agricultural and Life Sciences, The University of Tokyo, 1-1-1Bunkyo-Ku, YayoiTokyo, 113-8657 Japan; 3 Plant Molecular Research Center, Chiba University, Chiba, 260-0856 Japan

**Keywords:** Antitumor drugs, Controlled environment, Cultivation method, Medicinal plant production, Monoterpenoid indole alkaloids

## Abstract

*Ophiorrhiza pumila* is a medicinal plant that grows in subtropical forests and produces camptothecin (CPT). To determine an optimal harvest time of *O. pumila* in a plant factory with artificial light (PFAL), we investigated the CPT distribution in each organ and at the developmental stage and estimated the annual CPT production. For this study, the *O. pumila* plants were grown in controlled environments (16 h light period, photosynthetic photon flux density of 100 μmol m^−2^ s^−1^ under white light-emitting diode lamps, air temperature of 28 °C, relative humidity of 80%, and CO_2_ concentration of 1000 μmol mol^−1^). First, the stem, root, and seed pod had higher CPT contents than the leaves, flower, and ovary. The optimal harvest time of *O. pumila* in a PFAL was 63 days after transplanting (DAT), because the CPT content in the whole organs was the highest at the seed-ripening stage. Second, based on these results, the estimated annual CPT production of *O. pumila* cultivated in a PFAL was 380 mg m^−2^ y^−1^ (63 DAT). This value was 4.3 times greater than the annual CPT production by *Camptotheca acuminata* in a greenhouse. We concluded that the CPT production by *O. pumila* in a PFAL throughout the year has many advantages, although the demand for electrical energy was high compared to that of *Camptotheca acuminata* in a greenhouse.

## Introduction

**Fig. 1:**
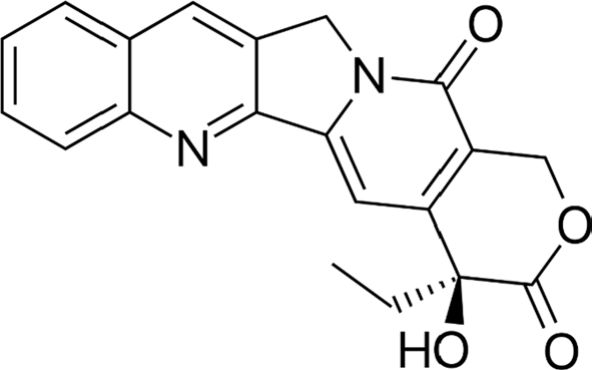
Chemical structure of the camptothecin

Camptothecin (Fig. 1) is a plant-based monoterpenoid indole alkaloid used as a raw material in antitumor drugs because of its inhibitory activity against DNA topoisomerase I [1, 2]. The semi-synthetic derivatives of CPT, topotecan and irinotecan have been employed worldwide as clinical antitumor agents against lungs, cervix, ovaries, and colon cancers [3–5]. The annual CPT production by plants globally is only 600 kg; thus, it cannot meet the world market's estimated demand of about 3000 kg per year [6]. Despite the high demand for CPT for use in antitumor drugs, CPT has been extracted mainly from two arboreous plants, *Camptotheca acuminata* and *Nothapodytes foetida* (Olacaceae), which have a slow growth rate and low plant productivity and require wide cultivation area and high photosynthetic photon flux density (PPFD) [2, 7]. Therefore, it is necessary to find an artificial synthesis method or an alternative medicinal plant species to produce CPT instead of the arboreous plants mentioned above.

Recently, growing attention has been given to CPT production by *Ophiorrhiza* species in the Rubiaceae family [8]. *Ophiorrhiza pumila* is one of the most important herbaceous perennial plants. It grows in mountainous, moist, and shady habitats in subtropical forests in the Ryukyu Islands in southern Japan, Taiwan, southern China, northern Vietnam, and the Philippines [9]. Asano et al. [10] established methods for hairy root cultures of genus *Ophiorrhiza* plants (*O. pumila*, *O. liukiuensis, and O. kuroiwai*) that produce CPT and related alkaloids, and the highest CPT production was obtained from the hairy root of *O. pumila*. Furthermore, *O. pumila* is a small-sized plant with a low PPFD requirement [11]; thus, it can be cultivated in limited spaces such as a greenhouse or plant factory with artificial light (PFAL) for CPT production instead of the arboreous plants that need to be cultivated in open-field.

A PFAL can control environmental conditions, including light, air temperature, humidity, and gas concentration, to optimize the growth of target plants. It also has several advantages compared to a greenhouse or open-field: no pesticides, reduction in water and fertilizer consumption by recycling, lower working hours and labor per unit yield, and reduction in product loss due to physical, chemical, and biological damage to the plant [12]. Additionally, plant productivity per unit area in a PFAL can increase if multi-layer cultivation shelves are used [13]. Therefore, a PFAL that can produce plants with stable quality throughout the year can be expected to have a high annual production of CPT by *O. pumila*. Previously, our research group established suitable environmental conditions for the growth of *O. pumila* in a PFAL [11, 14].

Many researchers have reported that the concentrations of secondary metabolites vary with plant organs and developmental stages [15–18]. Particularly, the concentration of alkaloids, which are defensive compounds, increased during the flowering or fruit development stage in *Fritillaria cirrhosa* [17] and *Sanguinaria canadensis* [15]. Yamazaki et al. [19] reported that young organs, including flower buds and the youngest leaves, had the highest CPT concentration among all organs of 6-month-old *O. pumila*. However, there is little information on the CPT concentration (mg g^−1^ DW) and yield (mg/plant) in each organ at the developmental stages of *O. pumila*. In this study, we investigated the CPT distribution in each organ and developmental stage of *O. pumila* to determine an optimal harvest time to obtain the maximum weight for annual CPT production in a PFAL. In addition, for the commercialization of CPT production, we evaluated the annual CPT production of *O. pumila* cultivated in a PFAL compared to that of *C. acuminata*, which is currently the main plant material used for the production of the antitumor agent CPT cultivated in greenhouses.

## Materials and methods

### Plant material and cultivation environmental conditions

Several tissue-cultured seedlings of *O. pumila* were provided by Professors Kazuki Saito and Mami Yamazaki (Chiba University, Japan). After propagation by tissue culture, plantlets were grown hydroponically, and the seeds were obtained in a PFAL at Chiba University in Matsudo, Japan. The seeds were sown in sterile Petri dishes containing Murashige and Skoog (MS) medium after seed surface-sterilization. 1 month after the seeds were sown, the seedlings were transplanted to a new MS medium in a sterile glass bottle.

The fresh weight of whole plants averaged 0.1 g after 3 weeks. The plants were transplanted to a urethane sponge as a substrate for hydroponics at a density of 178 plants m^−2^ in a PFAL and watered with Otsuka-A nutrient solution (OAT Agrio Co. Ltd., Japan) of 0.125 strength, which is widely used for leafy and fruit vegetable cultivation in Japan. The electrical conductivity (EC) of the standard concentration of Otsuka-A was 2.7 dS m^−1^ (including the EC of tap water); this concentration is referred to as the 1 strength in this study. The EC and pH of 0.125 strength were 0.6 dS m^−1^ and 6.5, respectively. The seedlings were covered with plastic wrap for maintaining a high relative humidity (RH; more than 98% approximately). To perform humidity acclimation, the RH near the seedlings was gradually decreased to 80% for 2 weeks.

After the humidity acclimation in RH of 80%, the fresh weight of whole plants averaged 0.3 g (*n* = 15), and the plants were transplanted to a culture panel, which was floated in a plastic container (18.6 L) filled with 9 L of nutrient solution at a density of 85 plants m^−2^. This date was 0 days after transplanting (DAT). The EC and pH of the nutrient solution were 0.9 dS m^−1^ (0.25 strength of the Otsuka-A nutrient solution) and 6.5, respectively, and the solution was replaced every 2 weeks [14]. After transplanting, the *O. pumila* plants were grown under suitable environmental conditions for growth [11, 14]; 16 h light period, PPFD of 100 ± 5 μmol m^−2^ s^−1^ under white light-emitting diode (LED) lamps (LDL40S-N/19/25, Panasonic Co., Ltd., Japan), air temperature of 28 °C, RH of 80%, and CO_2_ concentration of 1000 μmol mol^−1^. White LED lamps have become popular mass-produced lighting devices recently; therefore, many plant factories adopt them as a light source for saving energy and cost. In our preliminary experiment, no differences were found in the plant growth between the white LED and fluorescent lamps.

### Classification of organs

**Fig. 2:**
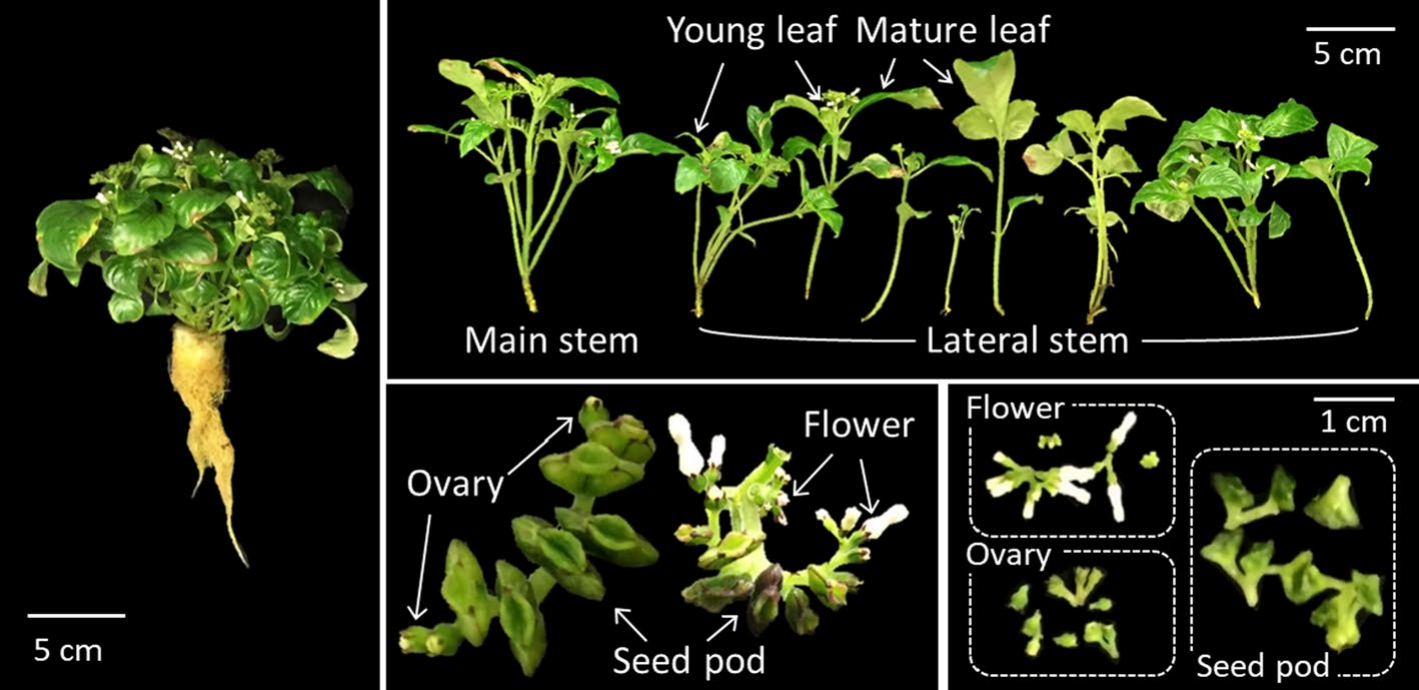
Classification of the plant organs of *Ophiorrhiza pumila* at 60 days after transplanting for analysis of camptothecin distribution in each organ. The plants were grown hydroponically under a 16 h light period, with a photosynthetic photon flux density of 100 ± 5 μmol m^−2^ s^−1^ under white light-emitting diode lamps, air temperature of 28 °C, RH of 80%, and CO_2_ concentration of 1000 μmol mol^−1^

Three mature plants bearing flowers and seed pods were randomly selected at 56 to 65 DAT and were separated into the root, young leaf, mature leaf, main stem, lateral stem, flower, ovary, and seed pod to investigate the CPT concentration distribution in each organ (Fig. 2). The leaves within 2 cm from the top layer of plants were classified as the young leaves, and the other leaves were classified as the mature leaves. The main stem was derived from the seed, and the other stems were classified as lateral stems. The flower and ovary included buds and immature seeds after the petals fell, respectively. The seed pod was the matured ovaries filled with seeds. The CPT and growth analysis methods are described as follows.

### Classification of developmental stage

**Fig. 3:**
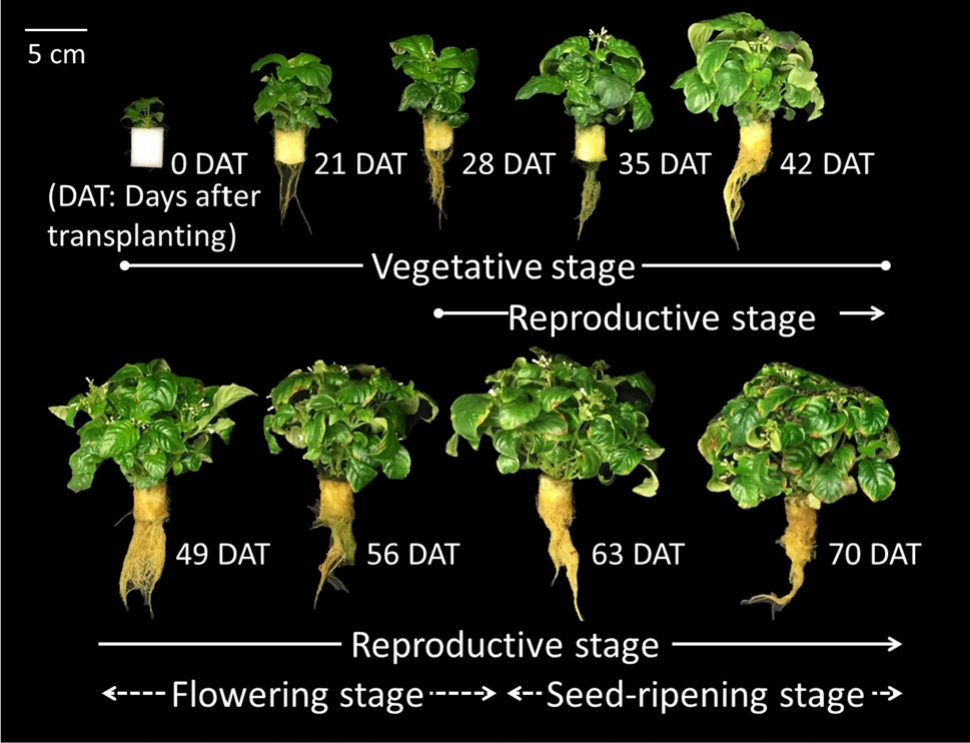
Developmental stages of *Ophiorrhiza pumila* grown under a 16 h light period, the photosynthetic photon flux density of 100 ± 5 μmol m^−2^ s^−1^ under white light-emitting diode lamps, air temperature of 28 °C, RH of 80%, and CO_2_ concentration of 1000 μmol mol^−1^

**Fig. 4:**
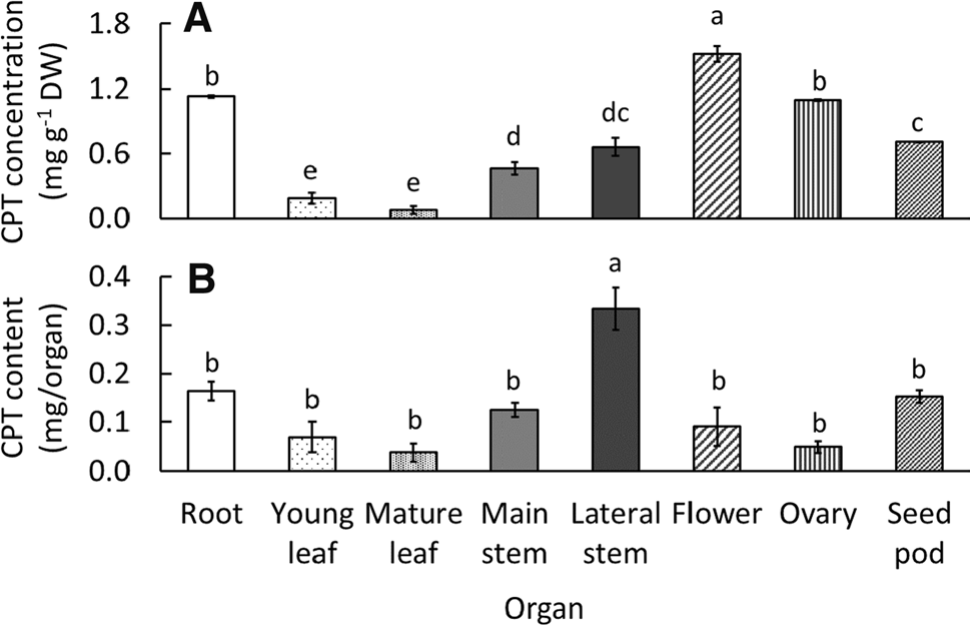
Camptothecin (CPT) concentration (**A**) and content (**B**) in each *Ophiorrhiza pumila* organ at 60 days after transplanting. The classification of organs and cultivation environments of *O. pumila* is described in Fig. 2. Vertical bars indicate standard error (*n* = 3). Different letters indicate significant differences among the organs at *P* < 0.05 by Tukey–Kramer’s test

The developmental stage was divided into vegetative (0 to 42 DAT) and reproductive stages (28 DAT and later) (Fig. 3). The reproductive stage was divided into the flowering stage (49 to 56 DAT) when most flowers bloom and the seed-ripening stage (57 DAT and later) when most seeds inside the seed pods are ripened. Based on the results of CPT distribution in each organ at 56 to 65 DAT (Fig. 4), the plant organs were separated simply into the root, stem (including main and lateral), leaf (including young and mature), and reproductive organ (including flower, ovary, and seed pod) to understand the transition of CPT concentration and content at each developmental stage.

### Growth characteristics

Plant heights, total leaf area, and the number of seed pods were measured weekly from 21 DAT. The plant height was measured using a ruler. Leaf area was calculated from photographs of the leaves using free imaging software (LIA 32 ver. 0.378) after all leaves were cut from the stems. After measuring the fresh weight of each organ, all organs were lyophilized for 24 h using a freeze dryer (FDU-1110, EYELA, Tokyo Rikakikai, Japan) to measure the dry weight. Following the measurement, the lyophilized samples were used for CPT analysis, as described below.

### CPT analysis

The CPT analysis was performed using the method described by Lee et al. [14]. Briefly, the extracted solution with methanol was analyzed using HPLC (LC-2010HT, Shimadzu Corporation, Kyoto, Japan). A solvent containing methanol and water in a ratio of 7:3 (w/v) was used for elution. A TSKgel ODS-80 V column (particle size 5 µm, 4.6 × 150 mm, Tosoh Corporation, Tokyo, Japan) was used for chromatography. The column temperature was maintained at 40 °C. Isocratic elution was performed at a flow rate of 1.0 mL min^−1^ for 20 min. The chromatogram was recorded at 365 nm wavelength. An HPLC grade of CPT (≥ 90%) (Sigma-Aldrich, USA) was purchased and used as a standard for identification and quantification. The standard curve for HPLC was constructed using five concentrations of CPT solution from 3.0 to 100.0 µg mL^−1^. The CPT concentration was calculated based on a dry weight basis.

### Annual CPT production estimation

The annual CPT production (mg m^−2^ y^−1^) by *O. pumila* at each growth stage (Fig. 3) in a PFAL was calculated by multiplying the number of harvests (year^−1^), plant density (plant m^−2^), and CPT content of the whole plant (mg/plant) in our results. The number of harvests was calculated by dividing 365 days by the cultivation period in each harvested stage. We assumed that the plant density was 178 plant m^−2^ at the seedling stage before transplanting until 28 DAT, or 85 plants m^−2^ after transplanting until harvest to prevent self- and mutual-shading of plants. The CPT content at harvest (mg/plant) is shown in Fig. 6E in this experiment.

We compared the estimated annual CPT production (mg m^−2^ y^−1^) by *O. pumila* in a PFAL to that by *C. acuminata* in a greenhouse reported by Vincent et al. [20]. For this estimation, we assumed that *O. pumila* was cultivated using a single-layer shelf in a PFAL, although considering multi-layers could have been possible. Vincent et al. [20] calculated the annual CPT yield from *C. acuminata* in a greenhouse. They reported that the maximum yield of CPT was 175 mg using approximately 2 m^2^ of greenhouse space and was obtained by harvesting young leaves twice at 6-week intervals for 12 weeks without destroying the trees.

### Statistical analysis

Data were statistically evaluated using one-way analysis of variance with the SPSS program for Windows (Version 24.0; SPSS Inc., Chicago, US). To investigate significant differences among plant organs and/or developmental stages, the means of measurement parameters were compared using the Tukey–Kramer test at *P* < 0.05.

## Results and discussion

### CPT concentration and content in each organ

#### CPT concentration

Among all organs, CPT concentration was higher in the order of the reproductive organ, root, stem, and leaves (Fig. 4A). The CPT concentration was the highest in flower (1.5 mg g^−1^ DW), and that in other reproductive organs, ovary, and seed pod (containing seeds), decreased with organ maturation. The CPT concentrations in the root and ovary were the second-highest (approximately 1.1 mg g^−1^ DW). The CPT concentration in the lateral stem, which contained more newly generated stems, was higher than that in the main stem. Although the CPT concentration in the leaves was the lowest significantly among all organs, it was higher in young leaves than in mature leaves. Among the same organs, the results showed that younger organs contained higher CPT concentrations.

Yamazaki et al. [19] reported results partially similar to ours, with the flower bud and youngest leaves in 6-month-old *O. pumila* having the highest CPT concentration among all organs. In addition, many studies on *C. acuminata* have also reported that young leaves and stems have higher CPT concentrations than mature leaves and stems [21–23]. Some studies suggested that young leaves and stems and reproductive organs accumulate CPT as a chemical defense mechanism against insects, pathogens, and other plants [19, 24].

The mechanism of CPT biosynthesis and translocation in *O. pumila* or other plants has not yet been identified. Liu [21] found that CPT accumulated primarily in glandular trichomes distributed in the young leaves and stems of *C. acuminata*. However, since there are no glandular trichomes in *C. acuminata* roots with high CPT concentration, Liu [21] suggested that CPT biosynthesis might occur in the roots, and then the molecule may be transported to young leaves and stems. In *O. pumila*, Yamazaki et al. [19] also reported that strictosidine synthase enzyme, a key enzyme of CPT biosynthesis, was detected in the roots and stems, but not in the leaves, suggesting that CPT may be synthesized in roots and stems. Our results on the distribution of CPT concentration among the organs were similar to the above reports, but further studies on CPT biosynthesis and translocation are needed.

#### CPT content

Among all organs, the lateral stem had the highest significant CPT content (0.3 mg/plant), because this part occupied approximately 18% of the dry weight of the whole plant (Fig. 4B). Next to the lateral stem, the CPT contents of the root, main stem, and seed pod were numerically higher than those of the other parts. Although the concentration of CPT in the flower was the highest among all organs, the content was lower than those of the stem and root, because the dry weight of the flower accounted for only 3% of the whole plant. In contrast, the young and mature leaves accounted for 46% of the total dry weight of the whole plant; however, their CPT contents were also low compared to the other organs because of the relatively low concentration of CPT. Therefore, we conclude that the stem (main and lateral stems), root, and seed pod are essential organs for CPT production from *O. pumila*.

Our research group reported that a light period of 16 h, as same as this experiment, promoted the flowering of *O. pumila* more than those of 8 and 12 h [11]. In addition, at 20 °C of the nutrient solution temperature, the CPT content of *O. pumila* was promoted because of the increase in the dry weight of the reproductive organ, root, and stem compared to that at 10 °C and 26 °C [14]. It is proposed that the environmental conditions that improve the growth of *O. pumila*, that is, the nutrient solution temperature of 20 °C, increased the total CPT content compared to this experiment without changing the CPT distribution among all organs. In conclusion, the high annual CPT production from whole *O. pumila* plants in a PFAL can be obtained through an increase in the dry weight of the essential organs (stem, root, and reproductive organ) of *O. pumila*.

### Growth characteristics, CPT concentration, and content at each developmental stage

#### Growth characteristics

**Fig. 5:**
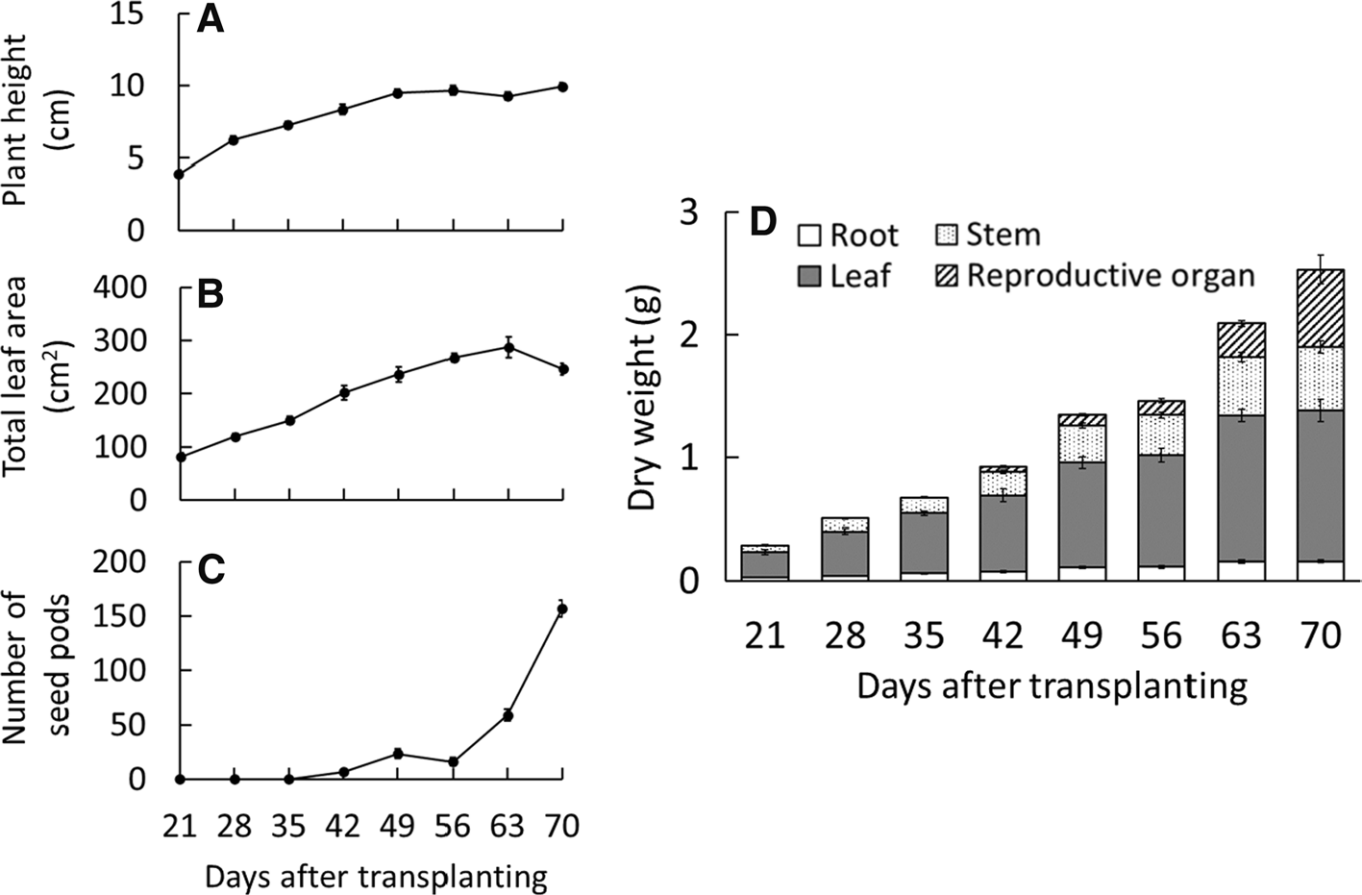
The transition of plant height (**A**), total leaf area (**B**), number of seed pods (**C**), and dry weight of the whole plant (**D**) of *Ophiorrhiza pumila* from 21 to 70 days after transplanting. The developmental stages of *O. pumila* and the cultivation environments are described in Fig. 3. Vertical bars indicate standard error (*n* = 5–8)

To determine the proper timing of plant harvest in a PFAL, it is important to understand the developmental stage of plants with a maximum yield of CPT, plant size, and leaf senescence at each stage. Throughout our experiment, plant heights peaked at approximately 10 cm at 49 DAT during the flowering stage (Fig. 5A). Because this plant with a short height (less than 30 cm) is suitable for efficient cultivation in a PFAL using multi-layer cultivation shelves [12], it was thought that the size of *O. pumila* was acceptable for the cultivation in a PFAL. In addition, *O. pumila* requires lower PPFDs compared to other leafy vegetables; therefore, the heat load from the light source of multi-layer cultivation shelves might be smaller. It is suggested that the density of *O. pumila* per unit cultivation area can be increased more than that of other leafy vegetables and that efficient CPT productivity can be achieved by using a PFAL.

The total leaf area continued to increase to 63 DAT, and until then, the aged leaves had fallen off (Fig. 5B). The number of seed pods increased sharply from 63 DAT, (Fig. 5C); thereafter, plant height and leaf area did not increase in the seed-ripening stage. The dry weights of the whole plants gradually increased throughout the experimental period (Fig. 5D). There is a possibility that dry weight will continue to increase even 70 DAT; however, since many aged or fallen leaves and flower petals may be infected by bacteria and filamentous fungi, it was suggested that the proper harvest timing of *O. pumila* for CPT production was 63 DAT, rather than 70 DAT.

Flowers, which contain a high CPT concentration at 60 DAT (Fig. 4), appeared from about 28 DAT (vegetative and/or reproductive stage), and the seed pods began to form after the petals fell from 49 DAT (flowering stage) (Fig. 5C and 5D). At 56 DAT (flowering stage), each plant contained approximately 14 seed pods (data not shown), but the seeds inside were immature. Especially from 63 DAT (seed-ripening stage), the dry weight of the reproductive organ increased sharply because of the maturation of the seeds inside the seed pods. At this stage, most of the flowers were transformed into seed pods, and many new flowers continued to appear from the lateral stems. Therefore, it was suggested that the plants after 63 DAT were fit for harvest when the dry weight of the reproductive organs begins to increase sharply. Regarding the plant aging problem mentioned above, it was concluded that the optimal harvest time was 63 DAT in this part for growth characteristics.

#### CPT concentration

**Fig. 6:**
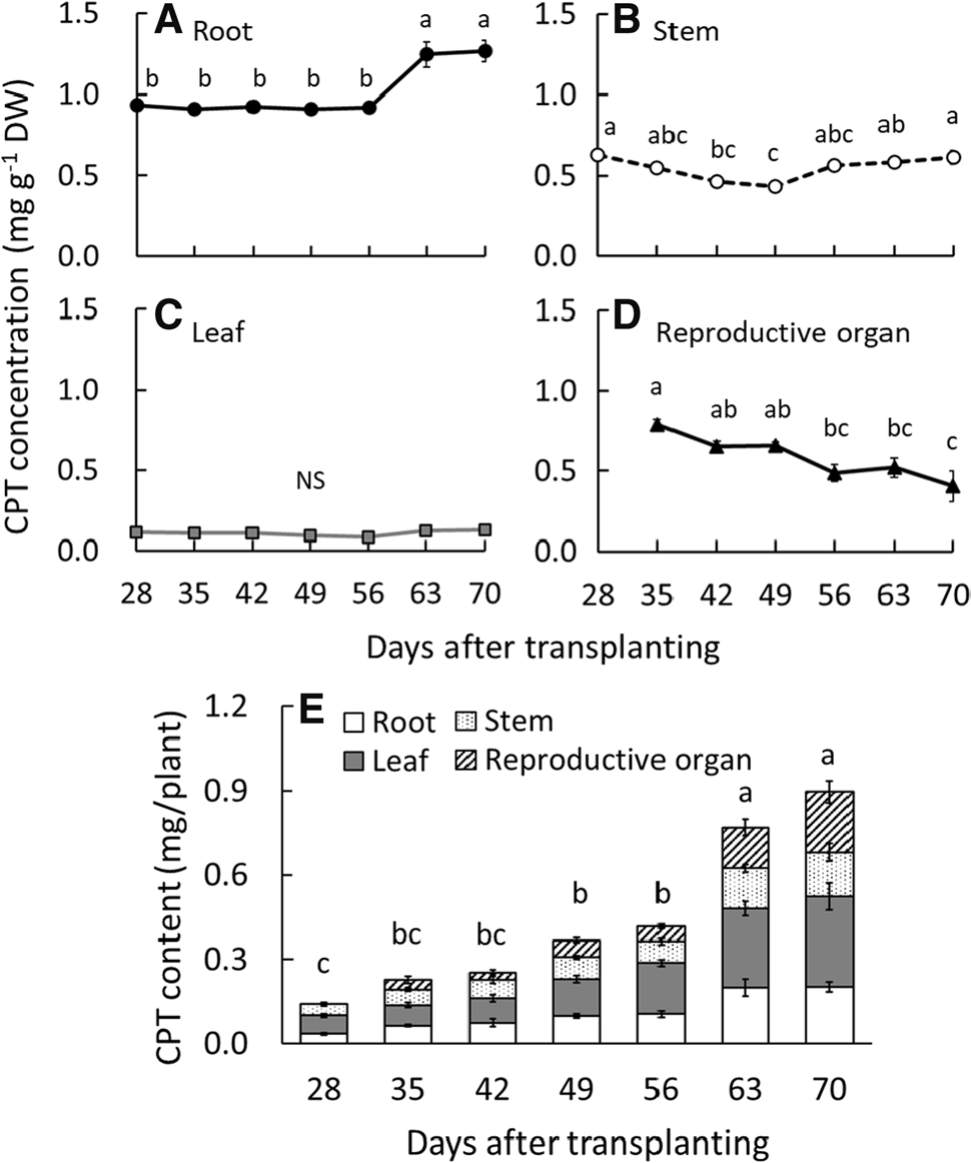
The transition of camptothecin (CPT) concentrations in the root (**A**), stem (**B**), leaf (**C**), and reproductive organ (**D**) and CPT content of the whole plant (**E**) of *Ophiorrhiza pumila* from 28 to 70 days after transplanting. The developmental stages of *O. pumila* and the cultivation environments are described in Fig. 3. Vertical bars indicate standard error (*n* = 5–6). Different letters indicate significant differences among the developmental stages at *P* < 0.05 by Tukey–Kramer’s test. (*NS* non-significant difference)

Among all organs, the root had the highest CPT concentrations throughout all stages (Fig. 6). At 63 DAT (seed-ripening stage), the CPT concentration in root was 35% higher than that at other stages (vegetative and flowering stages), and the difference was significant. The CPT concentrations in the stem gradually decreased by 49 DAT (flowering stage), and then continued to increase because of the generation of new lateral stems with high CPT concentrations (Fig. 6B). Conversely, the CPT concentration in the leaf, which was the lowest among all organs, was not significantly different across all developmental stages (Fig. 6C). The CPT concentrations in the reproductive organ continued to decrease after the flowering stages for the following reason: although the flower had the highest CPT concentration among all organs, in the reproductive organs, the proportion of seed pods with lower CPT concentration continued to increase (Fig. 6D). Therefore, it is considered that the seed-ripening stage of *O. pumila* is a suitable time to harvest the plants for CPT production, because the CPT concentration of root and stem increases at this stage.

#### CPT content

The CPT contents at the seed-ripening stage (63 and 70 DAT) were significantly and twice as high as those at the flowering stage (49 and 56 DAT) (Fig. 6E). The reason for this can be explained as follows: at the seed-ripening stage, 1) CPT concentrations increased in the roots and stems, and 2) the dry weight of the whole plant increased, particularly that of the reproductive organ. The CPT content continuously increased as dry weight increased because the CPT concentrations of all organs were maintained or increased with the progress of the developmental stages. In addition, since *O. pumila* plants have CPT in all organs, including roots and seeds, CPT production is possible through the harvest of whole plants without the need for organ separation. In conclusion, the seed-ripening stage (63 DAT) was optimal for harvesting to achieve maximum CPT yields from *O. pumila*. This is the first report on CPT accumulation transition throughout the growth stages of *O. pumila* and may contribute to the planned CPT production under a controlled environment in the future.

### Annual CPT production estimation

Annual CPT production by *O. pumila* cultivated in a PFAL increased with increasing cultivation period, except for 28 days (Table 1). During the cultivation period of 28 days, the plant density, number of harvests, and annual CPT production were higher than those at 35 to 56 DAT. Assuming that the annual CPT production is the same regardless of the cultivation period, a shorter cultivation period increases the labor costs for seedling production, transplanting, and cleaning of the cultivation spaces; thus, it is not economically advantageous. In contrast, the highest annual production of CPT was observed at 70 DAT. However, the proper harvest timing of *O. pumila* for CPT production seemed to be 63 DAT compared to 70 DAT, because of the aging problem at 70 DAT mentioned above. Therefore, we concluded that a cultivation period of 63 DAT was suitable for maximum annual CPT production by *O. pumila* cultivation in a PFAL.

**Table 1:** Estimation of annual CPT production by *Ophiorrhiza pumila* cultivated in a plant factory with artificial light

Cultivation period (days)	Number of harvests^a^ (year^−1^) (A)	Plant density^b^ (plants m^−2^) (B)	CPT content at harvest^c^ (mg/plant) (C)	CPT production^d^ (mg m^−2^ y^−1^)
28	13.0	178	0.14	324
35	10.4	85	0.23	203
42	8.7	85	0.25	185
49	7.4	85	0.37	233
56	6.5	85	0.42	232
63	5.8	85	0.77	380
70	5.2	85	0.90	398

^a^365 days was divided by the cultivation period (days)

^b^proper density to prevent the self-and mutual-shading

^c^The result in Fig. 6E in this paper

^d^CPT production = (A) × (B) × (C)

Vincent et al. [20] suggested that the annual CPT production from young leaves of *C. acuminata* in a greenhouse was 87.5 mg m^−2^ y^−1^ (Table 2). Because *C. acuminata* is a deciduous tree and the environment in a greenhouse is affected by the season outside, the optimal annual cultivation period for harvest is only 12 weeks. In addition, the initial CPT concentrations in *C. acuminata* ranged from 0.45 mg g^−1^ DW to 3.49 mg g^−1^ DW [20]. These high variations between individuals in *C. acuminata* in a greenhouse suggested that sustaining a stable CPT production is difficult for woody plants with a long cultivation period under unstable greenhouse environmental conditions compared to herbaceous plants in a PFAL. Conversely, from our result of CPT at 63 DAT, the annual CPT production of *O. pumila* cultivated in a PFAL was more than four times higher using a single shelf (380 mg m^−2^ y^−1^) than that of *C. acuminata* cultivated in a greenhouse.

**Table 2:** Annual CPT production by *Ophiorrhiza pumila* cultivated in a plant factory with artificial light (PFAL) and *Camptotheca acuminata* cultivated in a greenhouse

	Cultivation system	Harvest organ	Cultivation period (days)	Number of harvests (year^−1^)	CPT production (mg m^−2^ y^−1^)
*O. pumila*^a^	PFAL	Whole plant	63	5.8	380
*C. acuminata*^b^	Greenhouse	Young leaves	42	2.0	87.5

^a^it reported in this study

^b^Vincent et al. [20] reported the maximum CPT production of *C. acuminata* was 175 mg in 12 weeks using approximately 2 m^2^ of greenhouse space. They harvested the young leaves of *C. acuminata* two times repeatedly at a 6-week interval

In general, the resource use efficacy of plant production systems should be considered, including electricity, water, CO_2_, and labor for the next step. However, in total, the efficacy of annual CPT production of *O. pumila* cultivated in a PFAL was higher than that of *C. acuminata* cultivated in a greenhouse. In the near future, through the commercialization of *O. pumila* cultivation in a PFAL, high CPT production can be expected instead of arboreous plants.

## Conclusion

The stem, root, and seed pod had the highest CPT content among all organs of *O. pumila* cultivated in a PFAL. The seed-ripening stage at 63 DAT was the suitable harvest time to obtain the highest annual CPT production by *O. pumila* in a PFAL. This study suggests that CPT production by *O. pumila* cultivated in a PFAL meets the increasing demand for CPT as an antitumor agent instead of *C. acuminata* cultivated in an open-field or a greenhouse.
